# Tetra­phenyl­phospho­nium [μ_3_-(4-methyl­phen­yl)tellurolato]tris­[tetra­carbonyl­iron(0)]

**DOI:** 10.1107/S1600536813013056

**Published:** 2013-05-18

**Authors:** Yu-Long Li, Bin Xie, Li-Ke Zou

**Affiliations:** a School of Chemistry and Pharmaceutical Engineering, Institute of Functional Materials, Sichuan University of Science & Engineering, Zigong, Sichuan 643000, People’s Republic of China

## Abstract

In the anion of the title compound, (C_24_H_20_P)[Fe_3_(C_7_H_7_Te)(CO)_12_], each Fe^0^ atom is coordinated by four CO ligands and a Te atom, resulting in a trigonal–bipyramidal coordination environment. The Te atom is coordinated by a 4-methyl­phenyl group and the Fe^0^ atoms in a distorted tetra­hedral geometry. The average Te—Fe bond length is 2.574 (4) Å.

## Related literature

For related structures, see: Seyferth *et al.* (1985 ▶); Nicolet *et al.* (1999 ▶); Shieh & Shieh (1994 ▶). 
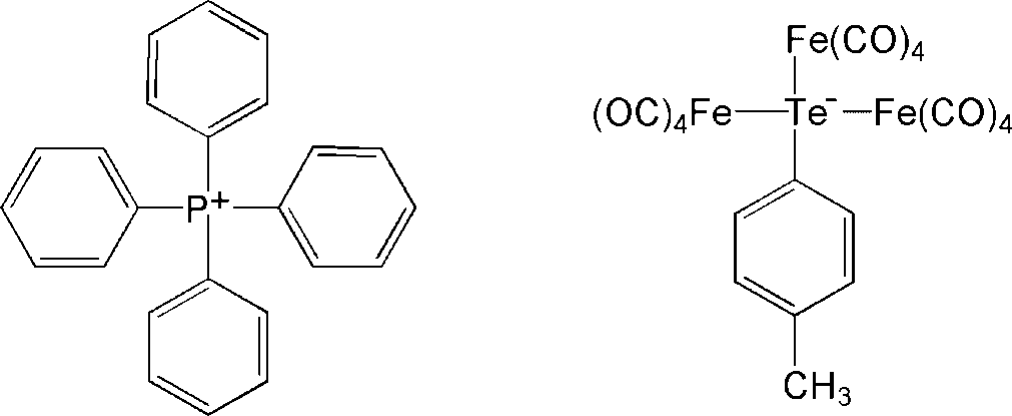


## Experimental

### 

#### Crystal data

(C_24_H_20_P)[Fe_3_(C_7_H_7_Te)(CO)_12_]
*M* *_r_* = 1061.77
Triclinic, 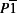

*a* = 12.367 (7) Å
*b* = 12.558 (6) Å
*c* = 13.626 (7) Å
α = 89.802 (17)°
β = 87.964 (13)°
γ = 86.312 (18)°
*V* = 2110.5 (19) Å^3^
*Z* = 2
Mo *K*α radiation
μ = 1.80 mm^−1^
*T* = 113 K
0.20 × 0.18 × 0.12 mm

#### Data collection

Rigaku Saturn724 CCD diffractometer
Absorption correction: multi-scan (*CrystalClear*; Rigaku/MSC, 2005 ▶) *T*_min_ = 0.715, *T*_max_ = 0.813
22136 measured reflections
9949 independent reflections
7690 reflections with *I* > 2σ(*I*)
*R*_int_ = 0.040

#### Refinement

*R*[*F* ^2^ > 2σ(*F* ^2^)] = 0.038
*wR*(*F* ^2^) = 0.068
*S* = 1.02
9949 reflections
542 parameters
H-atom parameters constrained
Δρ_max_ = 1.07 e Å^−3^
Δρ_min_ = −0.96 e Å^−3^

### 

Data collection: *CrystalClear* (Rigaku/MSC, 2005 ▶); cell refinement: *CrystalClear*; data reduction: *CrystalClear*; program(s) used to solve structure: *SHELXS97* (Sheldrick, 2008 ▶); program(s) used to refine structure: *SHELXL97* (Sheldrick, 2008 ▶); molecular graphics: *SHELXTL/PC* (Sheldrick, 2008 ▶); software used to prepare material for publication: *CrystalStructure* (Rigaku/MSC, 2005 ▶).

## Supplementary Material

Crystal structure: contains datablock(s) I, global. DOI: 10.1107/S1600536813013056/im2431sup1.cif

Structure factors: contains datablock(s) I. DOI: 10.1107/S1600536813013056/im2431Isup2.hkl

Additional supplementary materials:  crystallographic information; 3D view; checkCIF report

## Figures and Tables

**Fig. 1.**
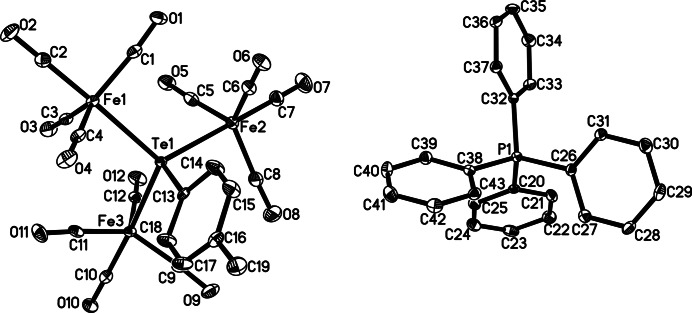
Molecular structure of the title compound, showing 30% probability displacement ellipsoids. H atoms have been omitted for clarity.

**Fig. 2.**
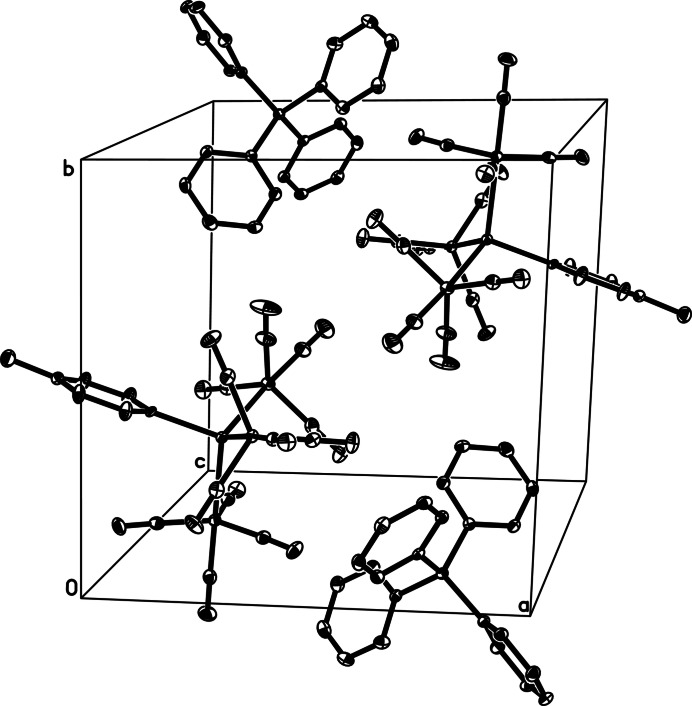
Packing diagram of the title compound.

## supplementary crystallographic information

### Comment

Fe/S cluster complexes containing µ-CO ligands have received considerable attention owing to their unique strucutre and particularly their apparent close relationship to the active site of [FeFe] hydrogenases (Seyferth *et al.*, 1985; Nicolet *et al.*, 1999). Herein, we report the synthesis and crystal struture of the title compound (Fig. 1). Each iron atom is coordinated by four CO ligands and Te1 resulting in a trigonal bipyramidal coordination environment. Tellurium is bonded to the 4-methylphenyl group and the iron atoms in a tetrahedral coordination mode. The average Te—Fe bond distances is 2.574 (1) Å is comparable to those found in the related structure [Et_4_N][BrFe_2_O_6_Te_2_] (Fe(1)—Te(1) = 2.524 (3) Å, Shieh & Shieh, 1994).

### Experimental

The reaction was carried out under nitrogen atmosphere. Te (0.128 g, 1.0 mmol) and *p*-tolylmagnesium bromide (1.0 ml, 1.0 *M* in THF) were added to THF (30 ml) in a Schlenk flask and stirred for 1.0 h at room temperature. Fe_3_(CO)_12_ (0.504 g, 1.0 mmol) was then added. After stirring for 0.5 h, Ph_4_PBr (0.420 g, 1.0 mmol) was added, the resulting mixture was stirred for 12 h, then volatiles were removed and the title compound was recrystallized from CH_2_Cl_2_/hexane (*v*/*v* = 1:1) to afford crystals. Yield: 10%. Anal. Calcd (%) for C_43_H_27_Fe_3_O_12_PTe (Mr = 1061.78): C, 48.64; H, 2.56; Found (%): C, 48.52; H, 2.61.

### Refinement

All H atoms were placed in geometrically idealized positions and constrained to ride on their parent atoms, with phenyl and methyl C—H distances of 0.95 and 0.98 Å, respecively. *U*_iso_(H) values were set to 1.2 (phenyl) and 1.5*U*_eq_(C) (methyl).

### Crystal data

**Table:**

(C_24_H_20_P)[Fe_3_(C_7_H_7_Te)(CO)_12_]	*Z* = 2
*M**_r_* = 1061.77	*F*(000) = 1052
Triclinic, *P*1	*D*_x_ = 1.671 Mg m^−^^3^
Hall symbol: -P 1	Mo *K*α radiation, λ = 0.71073 Å
*a* = 12.367 (7) Å	Cell parameters from 7686 reflections
*b* = 12.558 (6) Å	θ = 1.5–28.0°
*c* = 13.626 (7) Å	µ = 1.80 mm^−^^1^
α = 89.802 (17)°	*T* = 113 K
β = 87.964 (13)°	Prism, colorless
γ = 86.312 (18)°	0.20 × 0.18 × 0.12 mm
*V* = 2110.5 (19) Å^3^	

### Data collection

**Table:**

Rigaku Saturn724 CCD diffractometer	9949 independent reflections
Radiation source: rotating anode	7690 reflections with *I* > 2σ(*I*)
Multilayer monochromator	*R*_int_ = 0.040
Detector resolution: 14.22 pixels mm^-1^	θ_max_ = 27.9°, θ_min_ = 1.5°
ω and φ scans	*h* = −16→16
Absorption correction: multi-scan (*CrystalClear*; Rigaku/MSC, 2005)	*k* = −16→14
*T*_min_ = 0.715, *T*_max_ = 0.813	*l* = −17→17
22136 measured reflections	

### Refinement

**Table:**

Refinement on *F*^2^	Primary atom site location: structure-invariant direct methods
Least-squares matrix: full	Secondary atom site location: difference Fourier map
*R*[*F*^2^ > 2σ(*F*^2^)] = 0.038	Hydrogen site location: inferred from neighbouring sites
*wR*(*F*^2^) = 0.068	H-atom parameters constrained
*S* = 1.02	*w* = 1/[σ^2^(*F*_o_^2^) + (0.018*P*)^2^] where *P* = (*F*_o_^2^ + 2*F*_c_^2^)/3
9949 reflections	(Δ/σ)_max_ = 0.005
542 parameters	Δρ_max_ = 1.07 e Å^−^^3^
0 restraints	Δρ_min_ = −0.96 e Å^−^^3^

### Special details

**Table:**

Geometry. All e.s.d.'s (except the e.s.d. in the dihedral angle between two l.s. planes) are estimated using the full covariance matrix. The cell e.s.d.'s are taken into account individually in the estimation of e.s.d.'s in distances, angles and torsion angles; correlations between e.s.d.'s in cell parameters are only used when they are defined by crystal symmetry. An approximate (isotropic) treatment of cell e.s.d.'s is used for estimating e.s.d.'s involving l.s. planes.
Refinement. Refinement of *F*^2^ against ALL reflections. The weighted *R*-factor *wR* and goodness of fit *S* are based on *F*^2^, conventional *R*-factors *R* are based on *F*, with *F* set to zero for negative *F*^2^. The threshold expression of *F*^2^ > σ(*F*^2^) is used only for calculating *R*-factors(gt) *etc*. and is not relevant to the choice of reflections for refinement. *R*-factors based on *F*^2^ are statistically about twice as large as those based on *F*, and *R*- factors based on ALL data will be even larger.

### Fractional atomic coordinates and isotropic or equivalent isotropic displacement parameters (Å^2^)

**Table:**

	*x*	*y*	*z*	*U*_iso_*/*U*_eq_	
Te1	0.691679 (15)	0.758534 (15)	0.266548 (13)	0.01634 (5)	
Fe1	0.89752 (3)	0.77509 (3)	0.27611 (3)	0.01900 (10)	
Fe2	0.60360 (4)	0.69476 (3)	0.42882 (3)	0.02299 (11)	
Fe3	0.64378 (3)	0.64786 (3)	0.11779 (3)	0.01782 (10)	
P1	0.00221 (6)	0.76504 (6)	0.75476 (5)	0.01717 (17)	
O1	0.88942 (18)	0.78268 (17)	0.49180 (15)	0.0343 (6)	
O2	1.13180 (19)	0.79072 (19)	0.27970 (18)	0.0438 (6)	
O3	0.93297 (18)	0.56280 (17)	0.18610 (16)	0.0358 (6)	
O4	0.87619 (18)	0.96158 (18)	0.14596 (17)	0.0397 (6)	
O5	0.7700 (2)	0.51627 (18)	0.42636 (16)	0.0406 (6)	
O6	0.65189 (19)	0.88384 (18)	0.54154 (16)	0.0389 (6)	
O7	0.5086 (2)	0.6040 (2)	0.60572 (18)	0.0514 (7)	
O8	0.3964 (2)	0.6788 (3)	0.33252 (19)	0.0706 (10)	
O9	0.42183 (19)	0.74524 (18)	0.11968 (17)	0.0396 (6)	
O10	0.60419 (18)	0.52660 (16)	−0.05756 (15)	0.0322 (6)	
O11	0.81703 (19)	0.74332 (18)	0.00161 (15)	0.0378 (6)	
O12	0.68476 (19)	0.44626 (17)	0.22563 (16)	0.0370 (6)	
C1	0.8880 (2)	0.7808 (2)	0.4075 (2)	0.0249 (7)	
C2	1.0394 (3)	0.7854 (2)	0.2783 (2)	0.0278 (7)	
C3	0.9171 (2)	0.6449 (2)	0.2214 (2)	0.0242 (7)	
C4	0.8830 (2)	0.8892 (3)	0.1975 (2)	0.0261 (7)	
C5	0.7045 (3)	0.5864 (3)	0.4247 (2)	0.0289 (8)	
C6	0.6334 (3)	0.8114 (3)	0.4946 (2)	0.0288 (8)	
C7	0.5452 (3)	0.6398 (3)	0.5365 (2)	0.0330 (8)	
C8	0.4788 (3)	0.6861 (3)	0.3673 (2)	0.0387 (9)	
C9	0.5090 (3)	0.7069 (2)	0.1223 (2)	0.0261 (7)	
C10	0.6190 (2)	0.5735 (2)	0.0122 (2)	0.0238 (7)	
C11	0.7505 (3)	0.7075 (2)	0.0494 (2)	0.0250 (7)	
C12	0.6694 (2)	0.5273 (2)	0.1866 (2)	0.0245 (7)	
C13	0.6250 (2)	0.9165 (2)	0.2345 (2)	0.0173 (6)	
C14	0.6116 (3)	0.9927 (2)	0.3076 (2)	0.0335 (8)	
H14	0.6314	0.9749	0.3726	0.040*	
C15	0.5699 (3)	1.0939 (2)	0.2870 (2)	0.0327 (8)	
H15	0.5621	1.1452	0.3382	0.039*	
C16	0.5388 (2)	1.1234 (2)	0.1926 (2)	0.0247 (7)	
C17	0.5556 (3)	1.0475 (2)	0.1210 (2)	0.0298 (8)	
H17	0.5372	1.0655	0.0556	0.036*	
C18	0.5986 (3)	0.9451 (2)	0.1406 (2)	0.0292 (8)	
H18	0.6098	0.8946	0.0888	0.035*	
C19	0.4883 (3)	1.2327 (2)	0.1721 (2)	0.0365 (8)	
H19A	0.4686	1.2373	0.1031	0.055*	
H19B	0.5403	1.2862	0.1853	0.055*	
H19C	0.4232	1.2460	0.2145	0.055*	
C20	−0.0707 (2)	0.6535 (2)	0.71677 (19)	0.0179 (6)	
C21	−0.1683 (2)	0.6338 (2)	0.7639 (2)	0.0269 (7)	
H21	−0.1978	0.6810	0.8138	0.032*	
C22	−0.2239 (3)	0.5452 (3)	0.7388 (2)	0.0307 (8)	
H22	−0.2914	0.5325	0.7707	0.037*	
C23	−0.1801 (3)	0.4762 (2)	0.6671 (2)	0.0299 (8)	
H23	−0.2173	0.4153	0.6503	0.036*	
C24	−0.0828 (3)	0.4949 (2)	0.6195 (2)	0.0283 (8)	
H24	−0.0536	0.4474	0.5698	0.034*	
C25	−0.0275 (2)	0.5836 (2)	0.6444 (2)	0.0236 (7)	
H25	0.0397	0.5963	0.6120	0.028*	
C26	−0.0972 (2)	0.8696 (2)	0.7888 (2)	0.0183 (6)	
C27	−0.1827 (2)	0.8945 (2)	0.7263 (2)	0.0246 (7)	
H27	−0.1860	0.8580	0.6656	0.030*	
C28	−0.2623 (2)	0.9723 (2)	0.7529 (2)	0.0281 (7)	
H28	−0.3203	0.9898	0.7107	0.034*	
C29	−0.2568 (3)	1.0245 (2)	0.8419 (2)	0.0287 (8)	
H29	−0.3117	1.0776	0.8607	0.034*	
C30	−0.1730 (3)	1.0004 (2)	0.9032 (2)	0.0285 (8)	
H30	−0.1702	1.0373	0.9637	0.034*	
C31	−0.0921 (2)	0.9225 (2)	0.8776 (2)	0.0212 (7)	
H31	−0.0343	0.9056	0.9203	0.025*	
C32	0.0834 (2)	0.7279 (2)	0.85719 (19)	0.0171 (6)	
C33	0.0495 (2)	0.6507 (2)	0.9234 (2)	0.0221 (7)	
H33	−0.0150	0.6157	0.9128	0.027*	
C34	0.1104 (3)	0.6253 (2)	1.0050 (2)	0.0266 (7)	
H34	0.0883	0.5717	1.0494	0.032*	
C35	0.2029 (2)	0.6774 (2)	1.0217 (2)	0.0263 (7)	
H35	0.2439	0.6606	1.0779	0.032*	
C36	0.2357 (2)	0.7546 (2)	0.9557 (2)	0.0234 (7)	
H36	0.2994	0.7904	0.9671	0.028*	
C37	0.1771 (2)	0.7798 (2)	0.8741 (2)	0.0209 (7)	
H37	0.2004	0.8325	0.8293	0.025*	
C38	0.0915 (2)	0.8065 (2)	0.65763 (19)	0.0178 (6)	
C39	0.1806 (2)	0.7382 (2)	0.6292 (2)	0.0236 (7)	
H39	0.1950	0.6730	0.6633	0.028*	
C40	0.2480 (3)	0.7657 (3)	0.5512 (2)	0.0289 (8)	
H40	0.3077	0.7186	0.5308	0.035*	
C41	0.2285 (3)	0.8614 (3)	0.5029 (2)	0.0316 (8)	
H41	0.2741	0.8791	0.4484	0.038*	
C42	0.1435 (3)	0.9315 (2)	0.5329 (2)	0.0266 (7)	
H42	0.1324	0.9987	0.5014	0.032*	
C43	0.0741 (2)	0.9031 (2)	0.6099 (2)	0.0219 (7)	
H43	0.0144	0.9504	0.6298	0.026*	

### Atomic displacement parameters (Å^2^)

**Table:**

	*U*^11^	*U*^22^	*U*^33^	*U*^12^	*U*^13^	*U*^23^
Te1	0.01614 (11)	0.01721 (11)	0.01579 (10)	−0.00058 (8)	−0.00307 (7)	−0.00083 (7)
Fe1	0.0173 (2)	0.0202 (2)	0.0198 (2)	−0.00153 (19)	−0.00379 (18)	0.00052 (17)
Fe2	0.0226 (3)	0.0266 (3)	0.0199 (2)	−0.0029 (2)	−0.00044 (19)	0.00009 (18)
Fe3	0.0196 (2)	0.0169 (2)	0.0172 (2)	−0.00067 (18)	−0.00419 (17)	−0.00139 (17)
P1	0.0166 (4)	0.0184 (4)	0.0164 (4)	0.0003 (3)	−0.0012 (3)	−0.0007 (3)
O1	0.0375 (15)	0.0433 (15)	0.0229 (13)	−0.0049 (11)	−0.0083 (11)	−0.0015 (10)
O2	0.0220 (14)	0.0529 (17)	0.0578 (17)	−0.0086 (12)	−0.0094 (12)	0.0114 (13)
O3	0.0358 (15)	0.0288 (14)	0.0419 (15)	0.0091 (11)	−0.0081 (11)	−0.0114 (11)
O4	0.0329 (15)	0.0355 (15)	0.0523 (16)	−0.0102 (11)	−0.0097 (12)	0.0227 (12)
O5	0.0505 (17)	0.0358 (15)	0.0338 (14)	0.0138 (13)	−0.0083 (12)	−0.0006 (11)
O6	0.0508 (17)	0.0374 (15)	0.0287 (13)	−0.0038 (12)	−0.0020 (11)	−0.0077 (11)
O7	0.0589 (19)	0.0588 (18)	0.0367 (15)	−0.0127 (14)	0.0124 (13)	0.0067 (13)
O8	0.0275 (17)	0.141 (3)	0.0466 (18)	−0.0261 (18)	−0.0046 (14)	0.0042 (18)
O9	0.0290 (15)	0.0432 (16)	0.0461 (15)	0.0111 (12)	−0.0155 (11)	−0.0121 (12)
O10	0.0366 (14)	0.0345 (14)	0.0261 (12)	−0.0049 (11)	−0.0041 (10)	−0.0121 (10)
O11	0.0463 (16)	0.0428 (15)	0.0258 (13)	−0.0200 (12)	0.0076 (12)	0.0010 (11)
O12	0.0557 (17)	0.0238 (14)	0.0312 (13)	0.0028 (12)	−0.0083 (12)	0.0037 (10)
C1	0.0196 (18)	0.0237 (18)	0.0317 (19)	−0.0018 (14)	−0.0059 (14)	−0.0020 (14)
C2	0.031 (2)	0.0255 (19)	0.0273 (18)	−0.0022 (15)	−0.0034 (15)	0.0033 (13)
C3	0.0202 (18)	0.0293 (19)	0.0233 (17)	0.0009 (14)	−0.0052 (13)	0.0032 (13)
C4	0.0176 (17)	0.030 (2)	0.0316 (18)	−0.0047 (14)	−0.0055 (14)	−0.0006 (14)
C5	0.038 (2)	0.028 (2)	0.0214 (17)	−0.0037 (16)	−0.0011 (15)	0.0017 (14)
C6	0.030 (2)	0.033 (2)	0.0238 (18)	−0.0021 (16)	−0.0001 (14)	−0.0022 (14)
C7	0.032 (2)	0.037 (2)	0.0293 (19)	−0.0058 (16)	0.0022 (16)	0.0002 (15)
C8	0.029 (2)	0.061 (3)	0.0265 (19)	−0.0095 (19)	0.0018 (16)	0.0038 (17)
C9	0.030 (2)	0.0238 (18)	0.0248 (17)	−0.0021 (15)	−0.0089 (14)	−0.0071 (13)
C10	0.0209 (17)	0.0219 (18)	0.0287 (18)	−0.0015 (14)	−0.0020 (14)	0.0003 (13)
C11	0.034 (2)	0.0257 (18)	0.0162 (16)	−0.0022 (15)	−0.0055 (14)	−0.0057 (13)
C12	0.0256 (19)	0.0279 (19)	0.0201 (16)	0.0009 (15)	−0.0046 (13)	−0.0074 (14)
C13	0.0113 (15)	0.0140 (16)	0.0261 (16)	0.0032 (12)	−0.0032 (12)	0.0011 (12)
C14	0.051 (2)	0.0256 (19)	0.0227 (18)	0.0107 (17)	−0.0064 (16)	−0.0038 (14)
C15	0.049 (2)	0.0210 (19)	0.0268 (18)	0.0114 (16)	−0.0013 (16)	−0.0059 (14)
C16	0.0188 (17)	0.0211 (18)	0.0343 (19)	0.0000 (14)	−0.0024 (14)	0.0043 (14)
C17	0.042 (2)	0.0213 (18)	0.0274 (18)	−0.0015 (15)	−0.0150 (15)	0.0055 (13)
C18	0.044 (2)	0.0194 (18)	0.0249 (17)	−0.0003 (15)	−0.0115 (15)	−0.0025 (13)
C19	0.038 (2)	0.030 (2)	0.041 (2)	0.0018 (16)	−0.0048 (17)	0.0037 (16)
C20	0.0180 (16)	0.0189 (16)	0.0167 (15)	0.0018 (13)	−0.0057 (12)	0.0002 (11)
C21	0.0259 (19)	0.034 (2)	0.0211 (17)	−0.0023 (15)	0.0013 (14)	−0.0084 (14)
C22	0.0251 (19)	0.040 (2)	0.0285 (18)	−0.0105 (16)	−0.0006 (14)	0.0002 (15)
C23	0.039 (2)	0.0277 (19)	0.0253 (18)	−0.0120 (16)	−0.0103 (15)	0.0010 (14)
C24	0.036 (2)	0.0248 (19)	0.0246 (17)	−0.0032 (15)	−0.0029 (15)	−0.0038 (13)
C25	0.0254 (18)	0.0257 (18)	0.0197 (16)	−0.0021 (14)	−0.0003 (13)	0.0003 (13)
C26	0.0179 (17)	0.0172 (16)	0.0194 (15)	0.0015 (13)	0.0012 (12)	0.0002 (12)
C27	0.0257 (19)	0.0254 (18)	0.0224 (17)	0.0015 (14)	−0.0011 (13)	−0.0022 (13)
C28	0.0179 (18)	0.034 (2)	0.0315 (19)	0.0045 (15)	−0.0039 (14)	0.0028 (14)
C29	0.0232 (19)	0.0237 (18)	0.038 (2)	0.0076 (14)	0.0042 (15)	−0.0005 (14)
C30	0.033 (2)	0.0241 (19)	0.0280 (18)	0.0014 (15)	0.0013 (15)	−0.0046 (14)
C31	0.0183 (17)	0.0233 (17)	0.0223 (16)	−0.0017 (13)	−0.0019 (13)	−0.0024 (12)
C32	0.0167 (16)	0.0176 (16)	0.0163 (15)	0.0040 (13)	−0.0019 (12)	−0.0036 (11)
C33	0.0204 (17)	0.0238 (18)	0.0225 (16)	−0.0020 (14)	−0.0040 (13)	0.0017 (13)
C34	0.033 (2)	0.0241 (18)	0.0227 (17)	−0.0004 (15)	−0.0017 (14)	0.0064 (13)
C35	0.0291 (19)	0.0281 (19)	0.0216 (17)	0.0041 (15)	−0.0104 (14)	−0.0010 (13)
C36	0.0181 (17)	0.0276 (19)	0.0247 (17)	−0.0001 (14)	−0.0040 (13)	−0.0046 (13)
C37	0.0204 (17)	0.0198 (17)	0.0224 (16)	0.0004 (13)	−0.0015 (13)	−0.0015 (12)
C38	0.0194 (17)	0.0188 (16)	0.0153 (14)	−0.0015 (13)	−0.0017 (12)	0.0004 (11)
C39	0.0239 (18)	0.0225 (18)	0.0242 (17)	0.0006 (14)	0.0003 (13)	0.0017 (13)
C40	0.0233 (19)	0.031 (2)	0.0320 (19)	0.0019 (15)	0.0075 (14)	−0.0012 (15)
C41	0.035 (2)	0.035 (2)	0.0251 (18)	−0.0067 (17)	0.0068 (15)	0.0031 (14)
C42	0.033 (2)	0.0200 (18)	0.0273 (18)	−0.0035 (15)	−0.0027 (14)	0.0065 (13)
C43	0.0210 (17)	0.0195 (17)	0.0249 (17)	0.0022 (13)	−0.0038 (13)	−0.0030 (12)

### Geometric parameters (Å, º)

**Table:**

Te1—C13	2.148 (3)	C19—H19C	0.9800
Te1—Fe3	2.5693 (11)	C20—C21	1.384 (4)
Te1—Fe1	2.5754 (14)	C20—C25	1.391 (4)
Te1—Fe2	2.5780 (12)	C21—C22	1.395 (4)
Fe1—C2	1.769 (4)	C21—H21	0.9500
Fe1—C4	1.790 (3)	C22—C23	1.382 (4)
Fe1—C1	1.791 (3)	C22—H22	0.9500
Fe1—C3	1.797 (3)	C23—C24	1.380 (4)
Fe2—C7	1.770 (3)	C23—H23	0.9500
Fe2—C6	1.784 (3)	C24—C25	1.392 (4)
Fe2—C5	1.786 (4)	C24—H24	0.9500
Fe2—C8	1.792 (4)	C25—H25	0.9500
Fe3—C10	1.765 (3)	C26—C31	1.388 (4)
Fe3—C9	1.780 (3)	C26—C27	1.401 (4)
Fe3—C11	1.790 (3)	C27—C28	1.381 (4)
Fe3—C12	1.794 (3)	C27—H27	0.9500
P1—C26	1.791 (3)	C28—C29	1.386 (4)
P1—C38	1.791 (3)	C28—H28	0.9500
P1—C32	1.791 (3)	C29—C30	1.372 (4)
P1—C20	1.801 (3)	C29—H29	0.9500
O1—C1	1.150 (3)	C30—C31	1.390 (4)
O2—C2	1.150 (4)	C30—H30	0.9500
O3—C3	1.142 (3)	C31—H31	0.9500
O4—C4	1.147 (4)	C32—C37	1.392 (4)
O5—C5	1.158 (4)	C32—C33	1.396 (4)
O6—C6	1.154 (3)	C33—C34	1.390 (4)
O7—C7	1.136 (4)	C33—H33	0.9500
O8—C8	1.148 (4)	C34—C35	1.380 (4)
O9—C9	1.153 (4)	C34—H34	0.9500
O10—C10	1.146 (3)	C35—C36	1.391 (4)
O11—C11	1.145 (3)	C35—H35	0.9500
O12—C12	1.155 (3)	C36—C37	1.373 (4)
C13—C18	1.372 (4)	C36—H36	0.9500
C13—C14	1.382 (4)	C37—H37	0.9500
C14—C15	1.371 (4)	C38—C43	1.382 (4)
C14—H14	0.9500	C38—C39	1.397 (4)
C15—C16	1.397 (4)	C39—C40	1.384 (4)
C15—H15	0.9500	C39—H39	0.9500
C16—C17	1.367 (4)	C40—C41	1.380 (4)
C16—C19	1.501 (4)	C40—H40	0.9500
C17—C18	1.389 (4)	C41—C42	1.378 (4)
C17—H17	0.9500	C41—H41	0.9500
C18—H18	0.9500	C42—C43	1.392 (4)
C19—H19A	0.9800	C42—H42	0.9500
C19—H19B	0.9800	C43—H43	0.9500
			
C13—Te1—Fe3	103.91 (8)	C16—C19—H19C	109.5
C13—Te1—Fe1	105.46 (8)	H19A—C19—H19C	109.5
Fe3—Te1—Fe1	112.05 (2)	H19B—C19—H19C	109.5
C13—Te1—Fe2	108.79 (8)	C21—C20—C25	119.5 (3)
Fe3—Te1—Fe2	112.82 (4)	C21—C20—P1	119.4 (2)
Fe1—Te1—Fe2	113.06 (3)	C25—C20—P1	121.0 (2)
C2—Fe1—C4	91.25 (14)	C20—C21—C22	120.5 (3)
C2—Fe1—C1	90.42 (14)	C20—C21—H21	119.8
C4—Fe1—C1	124.22 (14)	C22—C21—H21	119.8
C2—Fe1—C3	90.69 (14)	C23—C22—C21	119.4 (3)
C4—Fe1—C3	118.77 (14)	C23—C22—H22	120.3
C1—Fe1—C3	116.96 (14)	C21—C22—H22	120.3
C2—Fe1—Te1	178.03 (10)	C24—C23—C22	120.6 (3)
C4—Fe1—Te1	87.96 (10)	C24—C23—H23	119.7
C1—Fe1—Te1	91.52 (10)	C22—C23—H23	119.7
C3—Fe1—Te1	88.11 (10)	C23—C24—C25	119.9 (3)
C7—Fe2—C6	90.84 (15)	C23—C24—H24	120.1
C7—Fe2—C5	89.61 (15)	C25—C24—H24	120.1
C6—Fe2—C5	117.33 (15)	C20—C25—C24	120.1 (3)
C7—Fe2—C8	90.40 (16)	C20—C25—H25	120.0
C6—Fe2—C8	121.94 (16)	C24—C25—H25	120.0
C5—Fe2—C8	120.73 (16)	C31—C26—C27	120.3 (3)
C7—Fe2—Te1	175.13 (11)	C31—C26—P1	120.6 (2)
C6—Fe2—Te1	93.60 (10)	C27—C26—P1	119.1 (2)
C5—Fe2—Te1	86.58 (10)	C28—C27—C26	119.9 (3)
C8—Fe2—Te1	88.95 (11)	C28—C27—H27	120.0
C10—Fe3—C9	91.86 (14)	C26—C27—H27	120.0
C10—Fe3—C11	88.46 (13)	C27—C28—C29	119.4 (3)
C9—Fe3—C11	121.47 (15)	C27—C28—H28	120.3
C10—Fe3—C12	90.79 (14)	C29—C28—H28	120.3
C9—Fe3—C12	117.38 (14)	C30—C29—C28	120.8 (3)
C11—Fe3—C12	121.14 (14)	C30—C29—H29	119.6
C10—Fe3—Te1	176.36 (10)	C28—C29—H29	119.6
C9—Fe3—Te1	90.85 (9)	C29—C30—C31	120.6 (3)
C11—Fe3—Te1	88.05 (9)	C29—C30—H30	119.7
C12—Fe3—Te1	90.11 (10)	C31—C30—H30	119.7
C26—P1—C38	111.26 (13)	C26—C31—C30	118.9 (3)
C26—P1—C32	110.38 (13)	C26—C31—H31	120.5
C38—P1—C32	107.57 (14)	C30—C31—H31	120.5
C26—P1—C20	106.86 (14)	C37—C32—C33	119.8 (3)
C38—P1—C20	110.75 (13)	C37—C32—P1	120.3 (2)
C32—P1—C20	110.05 (13)	C33—C32—P1	119.9 (2)
O1—C1—Fe1	175.3 (3)	C34—C33—C32	119.7 (3)
O2—C2—Fe1	179.2 (3)	C34—C33—H33	120.2
O3—C3—Fe1	177.8 (3)	C32—C33—H33	120.2
O4—C4—Fe1	178.3 (3)	C35—C34—C33	120.3 (3)
O5—C5—Fe2	177.1 (3)	C35—C34—H34	119.9
O6—C6—Fe2	176.4 (3)	C33—C34—H34	119.9
O7—C7—Fe2	179.3 (4)	C34—C35—C36	119.6 (3)
O8—C8—Fe2	176.4 (3)	C34—C35—H35	120.2
O9—C9—Fe3	176.3 (3)	C36—C35—H35	120.2
O10—C10—Fe3	178.6 (3)	C37—C36—C35	120.8 (3)
O11—C11—Fe3	176.6 (3)	C37—C36—H36	119.6
O12—C12—Fe3	175.7 (3)	C35—C36—H36	119.6
C18—C13—C14	118.6 (3)	C36—C37—C32	119.8 (3)
C18—C13—Te1	120.9 (2)	C36—C37—H37	120.1
C14—C13—Te1	120.5 (2)	C32—C37—H37	120.1
C15—C14—C13	120.5 (3)	C43—C38—C39	119.5 (3)
C15—C14—H14	119.7	C43—C38—P1	122.0 (2)
C13—C14—H14	119.7	C39—C38—P1	118.5 (2)
C14—C15—C16	121.7 (3)	C40—C39—C38	119.8 (3)
C14—C15—H15	119.1	C40—C39—H39	120.1
C16—C15—H15	119.1	C38—C39—H39	120.1
C17—C16—C15	116.7 (3)	C41—C40—C39	120.1 (3)
C17—C16—C19	122.1 (3)	C41—C40—H40	119.9
C15—C16—C19	121.2 (3)	C39—C40—H40	119.9
C16—C17—C18	122.2 (3)	C42—C41—C40	120.6 (3)
C16—C17—H17	118.9	C42—C41—H41	119.7
C18—C17—H17	118.9	C40—C41—H41	119.7
C13—C18—C17	120.2 (3)	C41—C42—C43	119.5 (3)
C13—C18—H18	119.9	C41—C42—H42	120.3
C17—C18—H18	119.9	C43—C42—H42	120.3
C16—C19—H19A	109.5	C38—C43—C42	120.4 (3)
C16—C19—H19B	109.5	C38—C43—H43	119.8
H19A—C19—H19B	109.5	C42—C43—H43	119.8
			
C13—Te1—Fe1—C2	−92 (3)	C12—Fe3—C11—O11	99 (5)
Fe3—Te1—Fe1—C2	21 (3)	Te1—Fe3—C11—O11	−172 (5)
Fe2—Te1—Fe1—C2	150 (3)	C10—Fe3—C12—O12	1 (4)
C13—Te1—Fe1—C4	−25.31 (12)	C9—Fe3—C12—O12	93 (4)
Fe3—Te1—Fe1—C4	87.09 (10)	C11—Fe3—C12—O12	−88 (4)
Fe2—Te1—Fe1—C4	−144.06 (10)	Te1—Fe3—C12—O12	−176 (4)
C13—Te1—Fe1—C1	98.89 (12)	Fe3—Te1—C13—C18	−18.1 (3)
Fe3—Te1—Fe1—C1	−148.71 (10)	Fe1—Te1—C13—C18	99.9 (2)
Fe2—Te1—Fe1—C1	−19.86 (10)	Fe2—Te1—C13—C18	−138.6 (2)
C13—Te1—Fe1—C3	−144.19 (12)	Fe3—Te1—C13—C14	164.4 (2)
Fe3—Te1—Fe1—C3	−31.79 (10)	Fe1—Te1—C13—C14	−77.6 (2)
Fe2—Te1—Fe1—C3	97.06 (10)	Fe2—Te1—C13—C14	44.0 (2)
C13—Te1—Fe2—C7	152.3 (14)	C18—C13—C14—C15	1.8 (5)
Fe3—Te1—Fe2—C7	37.5 (14)	Te1—C13—C14—C15	179.4 (3)
Fe1—Te1—Fe2—C7	−90.9 (14)	C13—C14—C15—C16	0.6 (5)
C13—Te1—Fe2—C6	−52.05 (14)	C14—C15—C16—C17	−2.3 (5)
Fe3—Te1—Fe2—C6	−166.80 (11)	C14—C15—C16—C19	177.0 (3)
Fe1—Te1—Fe2—C6	64.75 (11)	C15—C16—C17—C18	1.7 (5)
C13—Te1—Fe2—C5	−169.24 (13)	C19—C16—C17—C18	−177.6 (3)
Fe3—Te1—Fe2—C5	76.01 (11)	C14—C13—C18—C17	−2.4 (5)
Fe1—Te1—Fe2—C5	−52.45 (11)	Te1—C13—C18—C17	−180.0 (2)
C13—Te1—Fe2—C8	69.89 (14)	C16—C17—C18—C13	0.6 (5)
Fe3—Te1—Fe2—C8	−44.86 (12)	C26—P1—C20—C21	−35.4 (3)
Fe1—Te1—Fe2—C8	−173.31 (12)	C38—P1—C20—C21	−156.8 (2)
C13—Te1—Fe3—C10	92.7 (16)	C32—P1—C20—C21	84.4 (3)
Fe1—Te1—Fe3—C10	−20.6 (16)	C26—P1—C20—C25	148.0 (2)
Fe2—Te1—Fe3—C10	−149.6 (16)	C38—P1—C20—C25	26.7 (3)
C13—Te1—Fe3—C9	−45.43 (13)	C32—P1—C20—C25	−92.2 (3)
Fe1—Te1—Fe3—C9	−158.79 (10)	C25—C20—C21—C22	−0.5 (5)
Fe2—Te1—Fe3—C9	72.23 (11)	P1—C20—C21—C22	−177.2 (3)
C13—Te1—Fe3—C11	76.03 (13)	C20—C21—C22—C23	0.8 (5)
Fe1—Te1—Fe3—C11	−37.33 (10)	C21—C22—C23—C24	−0.8 (5)
Fe2—Te1—Fe3—C11	−166.31 (10)	C22—C23—C24—C25	0.6 (5)
C13—Te1—Fe3—C12	−162.81 (12)	C21—C20—C25—C24	0.3 (5)
Fe1—Te1—Fe3—C12	83.83 (10)	P1—C20—C25—C24	176.9 (2)
Fe2—Te1—Fe3—C12	−45.15 (10)	C23—C24—C25—C20	−0.4 (5)
C2—Fe1—C1—O1	−18 (3)	C38—P1—C26—C31	−108.6 (2)
C4—Fe1—C1—O1	−110 (3)	C32—P1—C26—C31	10.7 (3)
C3—Fe1—C1—O1	73 (3)	C20—P1—C26—C31	130.4 (2)
Te1—Fe1—C1—O1	161 (3)	C38—P1—C26—C27	74.0 (3)
C4—Fe1—C2—O2	−141 (19)	C32—P1—C26—C27	−166.6 (2)
C1—Fe1—C2—O2	95 (19)	C20—P1—C26—C27	−47.0 (3)
C3—Fe1—C2—O2	−22 (19)	C31—C26—C27—C28	0.3 (4)
Te1—Fe1—C2—O2	−74 (20)	P1—C26—C27—C28	177.6 (2)
C2—Fe1—C3—O3	−11 (7)	C26—C27—C28—C29	−0.4 (5)
C4—Fe1—C3—O3	80 (7)	C27—C28—C29—C30	0.5 (5)
C1—Fe1—C3—O3	−102 (7)	C28—C29—C30—C31	−0.4 (5)
Te1—Fe1—C3—O3	167 (7)	C27—C26—C31—C30	−0.2 (4)
C2—Fe1—C4—O4	35 (10)	P1—C26—C31—C30	−177.5 (2)
C1—Fe1—C4—O4	126 (10)	C29—C30—C31—C26	0.3 (4)
C3—Fe1—C4—O4	−57 (10)	C26—P1—C32—C37	−89.0 (2)
Te1—Fe1—C4—O4	−143 (10)	C38—P1—C32—C37	32.5 (3)
C7—Fe2—C5—O5	−36 (6)	C20—P1—C32—C37	153.3 (2)
C6—Fe2—C5—O5	55 (6)	C26—P1—C32—C33	87.4 (2)
C8—Fe2—C5—O5	−126 (6)	C38—P1—C32—C33	−151.0 (2)
Te1—Fe2—C5—O5	147 (6)	C20—P1—C32—C33	−30.3 (3)
C7—Fe2—C6—O6	17 (5)	C37—C32—C33—C34	−0.9 (4)
C5—Fe2—C6—O6	−73 (5)	P1—C32—C33—C34	−177.4 (2)
C8—Fe2—C6—O6	108 (5)	C32—C33—C34—C35	1.3 (4)
Te1—Fe2—C6—O6	−161 (5)	C33—C34—C35—C36	−0.9 (4)
C6—Fe2—C7—O7	−94 (30)	C34—C35—C36—C37	0.1 (4)
C5—Fe2—C7—O7	23 (30)	C35—C36—C37—C32	0.3 (4)
C8—Fe2—C7—O7	144 (30)	C33—C32—C37—C36	0.1 (4)
Te1—Fe2—C7—O7	62 (30)	P1—C32—C37—C36	176.6 (2)
C7—Fe2—C8—O8	5 (6)	C26—P1—C38—C43	−7.1 (3)
C6—Fe2—C8—O8	−86 (6)	C32—P1—C38—C43	−128.1 (2)
C5—Fe2—C8—O8	95 (6)	C20—P1—C38—C43	111.6 (2)
Te1—Fe2—C8—O8	−179 (100)	C26—P1—C38—C39	173.5 (2)
C10—Fe3—C9—O9	−36 (4)	C32—P1—C38—C39	52.5 (3)
C11—Fe3—C9—O9	53 (4)	C20—P1—C38—C39	−67.8 (3)
C12—Fe3—C9—O9	−128 (4)	C43—C38—C39—C40	−2.6 (4)
Te1—Fe3—C9—O9	141 (4)	P1—C38—C39—C40	176.8 (2)
C9—Fe3—C10—O10	108 (13)	C38—C39—C40—C41	1.3 (5)
C11—Fe3—C10—O10	−13 (13)	C39—C40—C41—C42	1.4 (5)
C12—Fe3—C10—O10	−134 (13)	C40—C41—C42—C43	−2.8 (5)
Te1—Fe3—C10—O10	−30 (14)	C39—C38—C43—C42	1.1 (4)
C10—Fe3—C11—O11	9 (5)	P1—C38—C43—C42	−178.2 (2)
C9—Fe3—C11—O11	−83 (5)	C41—C42—C43—C38	1.6 (5)
